# Triadic relations in healthcare: surveying physicians’ perspectives on generative AI integration and its role on empathy, the placebo effect and patient care

**DOI:** 10.3389/fpsyg.2025.1612215

**Published:** 2025-11-12

**Authors:** Vanda Faria, Nathan Goturi, Amanda Dynak, Cameron Talbert, Nicholas Pondelis, Marco Annoni, Charlotte Blease, Scott A. Holmes, Eric A. Moulton

**Affiliations:** 1 Brain and Eye Pain Imaging Lab, Pain and Affective Neuroscience Center, Department of Anesthesiology, Critical Care and Pain Medicine, Boston Children’s Hospital and Harvard Medical School, Boston, MA, United States; 2 Bioethics Research Unit - CNR Interdepartmental Center for Research Ethics and Integrity (CID-Ethics), National Research Council, Rome, Italy; 3 Participatory eHealth & Health Data Research Group, Department of Women's and Children's Health, Uppsala University, Uppsala, Sweden; 4 Digital Psychiatry, Beth Israel Deaconess Medical Center, Harvard Medical School, Boston, MA, United States; 5 Pediatric Pain Pathways Lab, Pain and Affective Neuroscience Center, Department of Anesthesiology, Critical Care and Pain Medicine, Boston Children’s Hospital and Harvard Medical School, Boston, MA, United States; 6 Department of Ophthalmology, Boston Children’s Hospital and Harvard Medical School, Boston, MA, United States

**Keywords:** generative AI, physicians’ perspectives, empathy, patient-physician relation, triadic relationship

## Abstract

**Background:**

The integration of generative artificial intelligence (genAI) tools into clinical practice and health care systems is reshaping modern healthcare, introducing technology as an active third participant in the evolving physician–patient–technology relationship. As these tools begin to play more prominent roles, understanding physicians’ perspectives is essential for guiding their ethical and effective use.

**Objective:**

This survey examined physicians’ use of genAI and their views on its potential impact on empathy, the physician–patient relationship, and psychobiological mechanisms such as the placebo and nocebo effects.

**Methods:**

A cross-sectional survey was distributed to 2,444 physicians at a major academic pediatric hospital in Boston (October 2024–February 2025). The survey included items on genAI use, perceptions of its clinical and relational impact, and associated concerns. A total of 319 (13%) completed responses were analyzed using descriptive and inferential statistics.

**Results:**

Within 2 years of the public release, 65.2% of respondents had used genAI tools, most commonly for administrative tasks like writing emails (55.8%) and documentation (31.3%). Use was more common among younger physicians and men. A majority believed genAI could improve patient care (60.7%) and increase time for direct interaction (65.7%). However, views were more divided regarding its ability to support empathetic care or influence placebo effects, with many physicians expressing neutrality or uncertainty. Notably, 50.8% agreed that genAI-human interactions could increase patient anxiety, indicating concern about potential nocebo effects. Perspectives on broader genAI adoption were mixed, with 30.6% expressing concern and 37.7% neutral.

**Conclusion:**

Physicians are rapidly adopting genAI tools, primarily for administrative use, while remaining cautious about its relational and psychological implications. These findings underscore the importance of addressing ethical concerns and supporting clinicians as they navigate this evolving triadic relationship between physician, patient, and genAI.

## Introduction

An evolving triadic relationship between physician, patient and technology is becoming increasingly central to modern healthcare, particularly with the rise of generative artificial intelligence (genAI) in healthcare. This shift marks a new era in medical practice, shaped not only by clinical expertise and patient experience, but also by the growing influence of genAI as an active participant in the physician–patient dynamic. Since the public release of the large language model (LLM)-based tool ChatGPT-3.5 in late 2022, followed by the more advanced ChatGPT-4 in 2023, the adoption of such tools in clinical settings has accelerated, bringing with it both transformative potential and complex challenges (Zhang and Kamel Boulos, 2023; Blease et al., 2024b; Fleurence et al., 2024; Torous and Blease, 2024; Foote et al., 2025; General Practitioners’ Experiences with Generative Artificial Intelligence in the UK: An Online Survey, 2025). These new generation chatbots, trained on vast datasets, function as advanced language models, capable of generating contextually relevant responses. Unlike traditional search engines, they can engage in dialogue, summarize information efficiently, and retain “memory” across multiple prompts, making them suited for interactive applications in healthcare.

GenAI has demonstrated promising capabilities in supporting routine clinical tasks, such as summarizing patient records, drafting discharge summaries, and even assisting with diagnostic reasoning (Haupt and Marks, 2023; Kanjee et al., 2023; Sharma B. et al., 2023). Questions also arise regarding patients and healthcare professionals adoption (Wutz et al., 2023). Although few studies have explored the uptake of genAI tools by patients, available evidence suggests that some are beginning to turn to these technologies and are deriving support from their use (Presiado et al., 2024; Siddals et al., 2024). However, as these generative models begin to occupy more patient-facing roles, new questions emerge around the communicative and relational dimensions of this care. The tone, framing, and delivery of information generated by genAI can shape patients’ emotional responses, sense of support or empathy, understanding and expectations of treatment. This growing role of genAI in emotionally sensitive areas of care has sparked debate about its capacity for empathy and compassionate responses (Morrow et al., 2023; Inzlicht et al., 2024). While critics argue that genAI lacks the moral reasoning, emotional nuance, and relational depth intrinsic to traditional caregiving (Perry, 2023; Akingbola et al., 2024), emerging research challenges the notion that empathy is an exclusively human capability (Ayers et al., 2023; Sharma A. et al., 2023; Kharko et al., 2024; Pan et al., 2024; Ovsyannikova et al., 2025). In settings where human contact is limited, genAI-powered systems have shown potential to simulate empathetic communication and even surpass humans in blinded trials (Ayers et al., 2023; Hatch et al., 2025). For instance, text-based mental health tools using genAI have demonstrated the ability to mimic emotionally attuned responses (Sharma A. et al., 2023), and early studies suggest these systems can detect affective states like pain and respond in ways that users perceive as caring (Cao et al., 2021). On the other hand, research also shows that while some users may increasingly turn to genAI chatbots for emotional support, these tools can fail to recognize or respond appropriately to psychological distress, particularly in crisis situations, raising serious safety and ethical concerns (De Freitas et al., 2024). The responses offered by chatbots may even cause harm to vulnerable patients (Lovens, 2025).

As genAI systems become more embedded in clinical workflows, physicians remain pivotal in shaping how these tools are implemented, framed, and ethically integrated into care. Their perceptions influence not only how genAI is adopted but also how patients experience genAI mediated care. While some clinicians view genAI as a valuable adjunct for reducing administrative burden and enhancing decision-making (Stroud et al., 2025), others express concern that it could erode the relational and humanistic foundations of care (Wang et al., 2023). Surveys of physicians across specialties highlight this ambivalence, valuing genAI’s efficiency and diagnostic potential, yet worrying about depersonalization, legal liability, and loss of professional autonomy (Blease et al., 2019; Scheetz et al., 2021; Emiroglu et al., 2022; Blease et al., 2024c; Dean et al., 2024; Stroud et al., 2025). A recent study combining social media analysis with an online survey found that while physicians see potential for genAI to support clinical work, their optimism is tempered by significant concern regarding job security, underlying the ambivalent attitudes that continue to characterize professional perspectives (Weber et al., 2024).

A critical but underexplored aspect of this shift is how genAI might influence psychobiological mechanisms like the placebo and nocebo effects, which are shaped by patients’ expectations, emotions, and perceptions of care quality (Annoni and Miller, 2016). Expectancy theory and contextual models of placebo effects highlight that verbal suggestions, clinician demeanor, and the therapeutic context can produce observable improvement, or worsen outcomes, through expectancy-driven neurobiological pathways (Wager and Atlas, 2015; Kirsch, 2018). In this context, physicians’ attitudes and the way they frame genAI play a key role. For example, positive communication and empathetic framing by clinicians have been shown that enhance placebo responses, while uncertainty or negative framing can elicit nocebo effects (Faria et al., 2017; Barnes et al., 2019; Smith et al., 2021). Emerging studies extending these insights into digital health, suggest that framing also shape responses to genAI systems (Kosch et al., 2023). Users who were told a genAI chatbot was designed to be caring rated it as more empathetic, trustworthy, and effective, even though its behavior remained the same (Pataranutaporn et al., 2023). Similarly, expectations about digital interventions, shaped by clinicians and context, can modulate users engagement and perceived support. These findings suggest that the way physicians present genAI, as empathetic, competent, and trustworthy versus impersonal or flawed, may influence patients’ expectations and, in turn, activate placebo or nocebo mechanisms that shape clinical outcomes. When it comes to trust, a core factor in genAI adoption, studies have highlighted that healthcare workers’ trust in genAI clinical decisions depends on perceived reliability, transparence and knowledge about the system (Markus et al., 2021; Afroogh et al., 2024; Goisauf et al., 2025). For clinicians, this raises practical and ethical questions: Can genAI genuinely contribute to empathetic care? Does its use risk diminish human connection? And how do physicians perceive genAI’s influence on patient expectations and its potential to shape therapeutic outcomes? For patients, genAI raises important questions about trustworthiness, perceptions of clinicians who use these tools, particularly for communication or for augmenting empathic engagement, and whether genAI itself can be seen as a source of support or empathy.

As genAI is more integrated in patient care, understanding physicians’ perspectives is critical. While most research focuses on genAI’s technical performance, far less is known about its relational and psychological dimensions of care such as perceived impact on empathy, trust, and expectation-driven mechanisms like the placebo and nocebo effects. Although some surveys have explored clinicians’ general views on genAI and its implications for communication and empathy, the potential role of genAI in shaping placebo and nocebo processes remains largely under investigated (Blease, 2025). This study addressed this gap by surveying physicians at a major academic pediatric hospital in Boston, exploring their experiences with genAI, and their views on its role in shaping human-centered aspects of care. Because prior research on clinicians’ perspectives on the topic is limited, this study adopts an exploratory approach. No *a priori* hypotheses were formulated, as theoretical frameworks have yet to be extended to genAI-mediated clinical contexts. We sought to generate insights into how physicians perceive genAI’s potential to support or undermine empathy, trust, and expectation related mechanisms in clinical practice. These insights are essential for guiding ethical, effective integration of genAI into clinical practice.

## Methods

### Survey development and piloting

The survey was designed and administered using REDCap, a secure online platform for managing surveys and databases, ensuring robust security, regulatory compliance, and automated data validation (Harris et al., 2019). Item generation was developed through a focus group discussion involving five expert clinicians and researchers from diverse fields, including psychology, placebo research, neuroscience, and bioethics. This group identified relevant domains and drafted items to capture physicians’ perspectives on genAI in clinical practice. All items were newly developed for this study. The resulting questions were clustered into five distinct thematic sections: (1) GenAI Usage Among Physicians: to assess the extent of genAI use in clinical practice, including the most commonly used tools and their applications; (2) Physicians’ Perceptions of genAI: to explore genAI’s potential to increase patient interaction time, support empathetic care, and enhance overall patient care; (3) GenAI and the Patient-Physician Relationship: to examine genAI’s role in supporting patient trust, strengthening the physician-patient relationship, increasing patient confidence in treatments, and potentially augmenting the placebo effect; (4) GenAI in Clinical Decision-Making and Patient Interaction: to focus on genAI’s potential to take on a more prominent role in clinical settings, including its ability to replace aspects of physician-patient interaction, assist in treatment selection, and engage directly with patients. It also assessed physicians’ perspectives on whether genAI-patient interactions could convey empathy, elicit placebo effects, or trigger the nocebo effect; and finally, (5) Concerns Regarding genAI Integration in Patient Care: to address physicians’ concerns about the integration of genAI technologies into patient care. The survey distinguished between empathy conveyed directly through genAI-patient interactions (eg., via chatbots) and empathy supported indirectly through physicians’ use of genAI as a tool in care delivery.

The survey was piloted with 10 experienced physicians from the Headache Clinic and Ophthalmology Department at BCH, who provided structured feedback on the clarity, comprehensibility, and relevance of the questions. While the participants generally found the survey straightforward and easy to complete, they also offered a small number of minor suggestions focused primarily on linguistic clarity, such as refining wording, adding examples, and improving user comfort. These comments collected through structured feedback, did not raise substantial concerns about the survey’s structure or overall design. Based on this input, minor revisions were incorporated into the final version, which comprised 18 questions, including demographic items. Hence, face validity of the instrument was established through expert review and piloting and revisions were incorporated to improve clarity and relevance. However, no formal psychometric validation, such as reliability testing or internal consistency analysis, was conducted. This is because the survey was intended to capture exploratory insights into physicians’ perspectives on genAI potential influence on placebo and nocebo responses, an aspect of clinical care that has not previously been systematically examined. The survey took an average of 5 min to complete; the full survey with instructions is available in the Supplementary material. The study was reviewed and approved by the Boston Children’s Hospital Institutional Review Board (IRB-P00048679). It was granted an exemption from requiring formal ethics approval, as it was classified as minimal risk.

### Participant recruitment and survey distribution

Physicians across all specialties at BCH were recruited via email to participate in the study. The recruitment email outlined the survey’s content, the study objectives, and participant’s rights. Physicians choosing to participate could access the survey through the link provided at the end of the email. No incentives were provided, and participation remained entirely voluntary and anonymous. Before beginning the survey, participants provided electronic written informed consent. Data collection occurred between October 2024 and February 2025. Eligibility was determined based on the following criteria: physicians holding a Doctor of Medicine (MD), Doctor of Osteopathic Medicine (DO), or Bachelor of Medicine, Bachelor of Surgery (MBBS) degree. The survey was distributed via individual email addresses (*n* = 2,444), which were obtained from BCH listservs with assistance from the Clinical Research Informatics team and the Human Resources team. Aside from the requirement that participants be practicing physicians at BCH, no additional exclusion criteria were applied when selecting email addresses. To encourage responses, three follow-up emails were sent to express appreciation and remind recipients about the survey. Of the 2,444 physicians initially contacted, 366 physicians (15%) returned their survey, a response rate comparable to previous placebo survey studies (Faria et al., 2023). However, 47 surveys were submitted blank and were thus excluded from the analysis, resulting in a final sample size of 319 (13%) usable surveys.

### Statistical analysis

Data were analyzed using SPSS version 22.0 software (SPSS Inc., Chicago, IL). Descriptive statistics, including percentages for categorical variables and means with standard deviations (SDs) for continuous variables, were reported. For most survey questions, response categories were merged, grouping positive responses (“Strongly Agree” and “Agree”) and negative responses (“Strongly Disagree” and “Disagree”). The percentages were reported for the response categories. Additionally, a Chi-Square test was performed to assess the association between gender (male vs. female) and genAI usage (user vs. non-user) and an independent samples t-test was conducted to compare age differences between genAI users and non-users. After each section of questions, participants had the opportunity to provide additional comments. These responses were categorized and summarized in a commentary table, while the original answers that formed the basis for these categories are presented in the Supplementary Tables 1–4.

## Results

### Demographics and work-related characteristics

Among the 319 participants, the majority identified as female (44.2%) or male (34.2%), while 21.7% chose not to disclose their gender by either selecting not listed above, leaving the field blank or explicitly opting not to disclose. Among them, one respondent specifically identified as a transgender woman. The average age of respondents was 46.8 ± 12.4 years, with a mean professional experience of 16.2 ± 11.7 years. The three most common specialties among participants were pediatrics (24.8%), anesthesiology (5%), and ophthalmology (4.7%). For a more detailed breakdown of participant characteristics, see Table 1.

**Table 1:** Demographics and work-related characteristics of physicians *N* = 319.

Characteristics	Value
Gender *N* (%)	
Woman	141 (44.20%)
Man	109 (34.17%)
Prefer not to say	7 (2.19%)
Not listed above	3 (0.94%)
No Response	59 (18.50%)
Age, yrs., mean (SD) [range]	46.8 (12.4) [25–84]
Years of Experience, mean (SD) [range]	16.2 (11.7) [0.5–57]
Specialty *N* (%)	
Pediatrics	79 (24.76%)
Anesthesiology	16 (5.02%)
Ophthalmology	15 (4.70%)
Neurology	14 (4.39%)
Cardiology	11 (3.45%)
Intensive care	9 (2.82%)
Emergency medicine	8 (2.51%)
Gastroenterology	8 (2.51%)
Hospital medicine	7 (2.19%)
Orthopedics	7 (2.19%)
Urology	6 (1.88%)
Psychiatry	6 (1.88%)
Primary Care	6 (1.88%)
Surgery	5 (1.57%)
Rheumatology	5 (1.57%)
Oncology	5 (1.57%)
Genetics	5 (1.57%)
Infectious disease	4 (1.25%)
Dermatology	4 (1.25%)
Neonatology	4 (1.25%)
Radiology	4 (1.25%)
Otolaryngology	3 (0.94%)
Hematology	3 (0.94%)
Endocrinology	3 (0.94%)
Allergy and Immunology	3 (0.94%)
Plastic Surgery	2 (0.63%)
Pathology	2 (0.63%)
Pulmonology	1 (0.31%)
Nephrology	1 (0.31%)
Pain Medicine	1 (0.31%)
Physical Medicine and Rehabilitation	1 (0.31%)
Pediatric Physical Medicine and Rehabilitation	1 (0.31%)
Sports Medicine	1 (0.31%)
Stem Cell Transplant	1 (0.31%)
Neurosurgery	1 (0.31%)
Pediatric Critical Care	1 (0.31%)
Obstetrics and Gynecology	1 (0.31%)
Maternal and Fetal Medicine	1 (0.31%)
Critical Care	1 (0.31%)
No response	63 (19.75%)

### Age, gender, and GenAI usage

**Figure 1:**
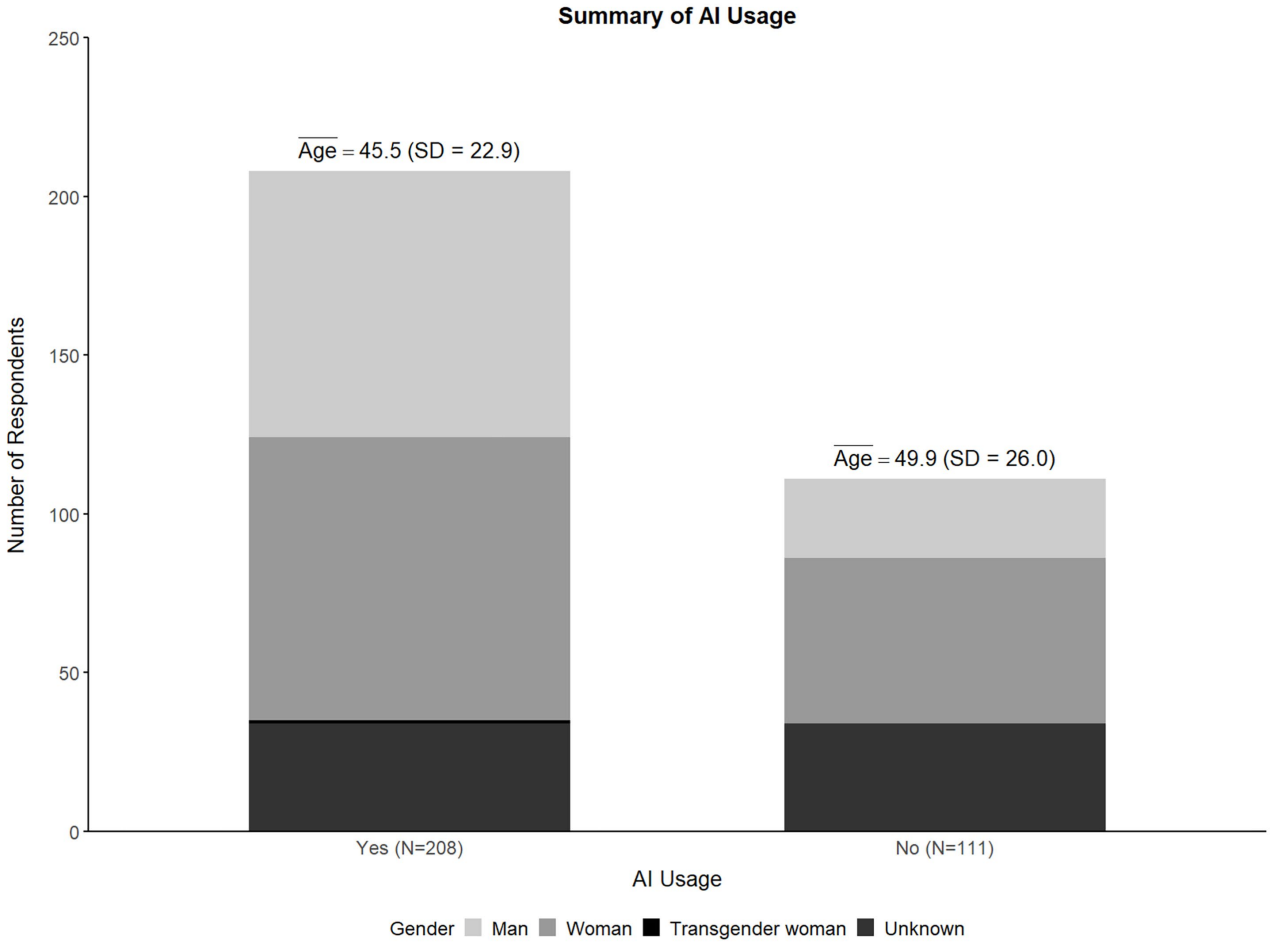
Demographics of genAI usage.

Figure 1 illustrates that nearly two-thirds (65.2%) of the surveyed physicians reported using genAI tools, while 34.8% indicated non-use. In terms of gender distribution, genAI usage was reported higher among men (77.1%) compared to women (63.1%), with 36.9% of women reporting non-use, a noticeably higher proportion than the 22.9% of men. This difference was statistically significant (*χ*^2^ (4) = 20.797, *p* < 0.001). In terms of age, genAI users were younger on average (*M* = 45.47, SD = 12.56) than non-users (*M* = 49.94, SD = 11.81). This age difference was also statistically significant (*t* (219) = 2.496, *p* = 0.013), with a mean difference of 4.47 years (95% CI [0.941, 7.996]) suggesting a small to moderate effect size (*d* = 0.362).

### GenAI usage in clinical practice

**Figure 2:**
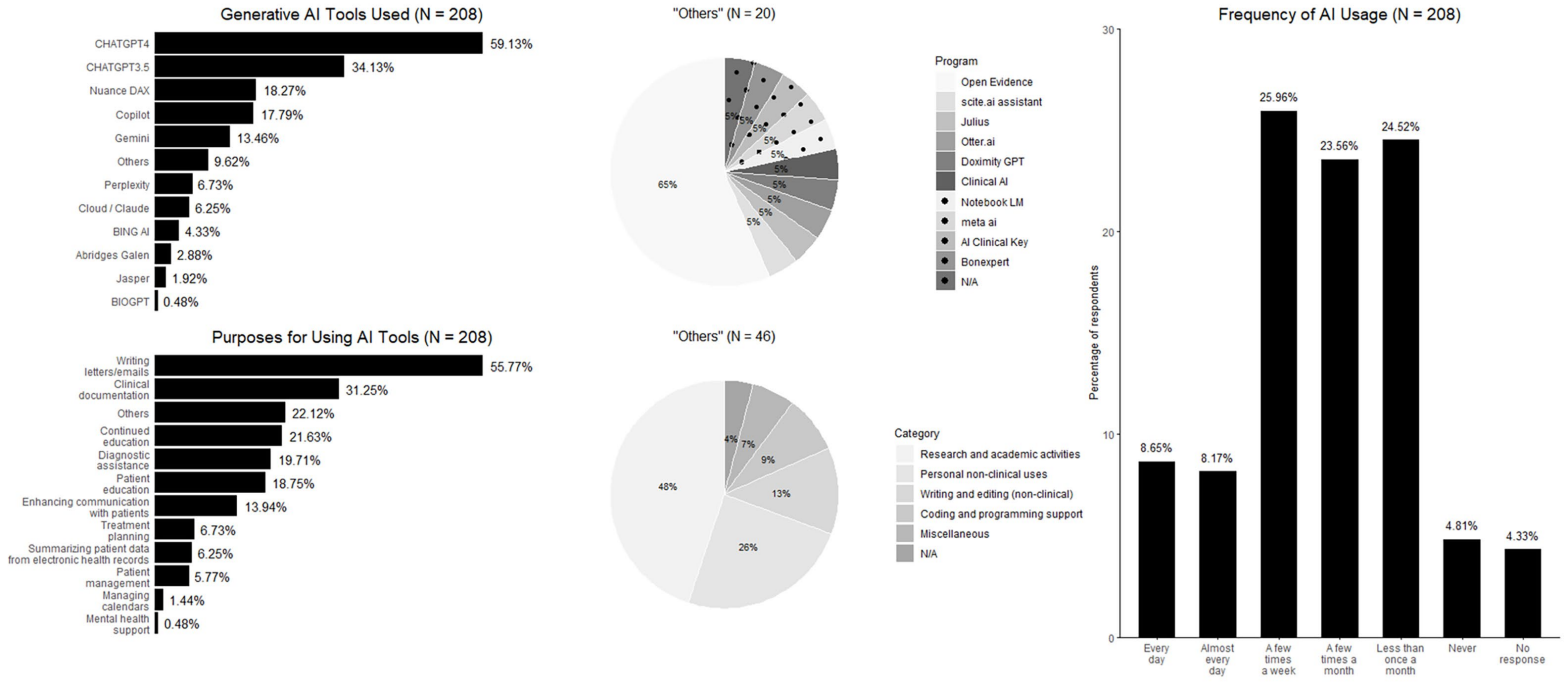
Experience and use of genAI in clinical settings.

This section examines the extent of genAI adoption in clinical practice, including the most frequently used tools and their primary applications. Among genAI users, the great majority (59.1%) reported the paywalled ChatGPT-4 as their most commonly used genAI-tool, followed by the free version of ChatGPT-3.5 (34.1%), and Nuance DAX (18.3%). Regarding the primary purposes of genAI usage, the most frequently reported applications were writing letters or emails (55.8%), followed by clinical documentation (31.3%), and continuing medical education (21.6%). In terms of usage frequency, 16.8% of surveyed physicians reported using genAI tools daily or almost daily, whereas one-quarter (25.9%) reported using them a few times per week. In contrast, nearly one-quarter (24.5%) reported genAI usage less than once a month, and 23.6% reported use a few times per month (Figure 2).

### Physicians’ perceptions of GenAI

**Figure 3:**
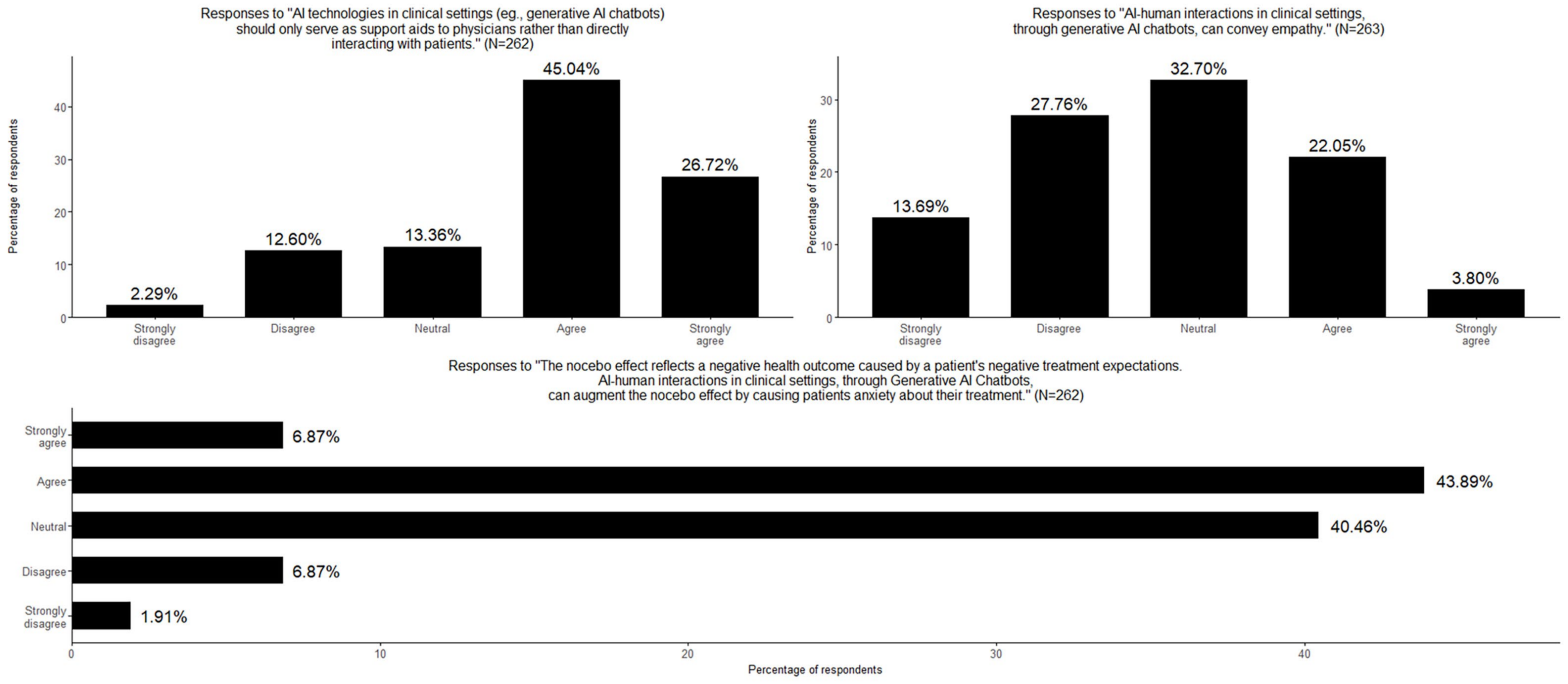
Physicians’ perspectives on genAI-patient interaction.

This section explores physicians’ perspectives on genAI’s potential to increase patient interaction time, support empathetic care, and enhance overall patient care. A substantial majority (65.8%) of physicians agreed that genAI will be a valuable tool for increasing the time available for patient interactions, whereas only 11.4% expressed disagreement (Table 2). Similarly, when considering the overall impact on patient care, the majority of respondents (60.7%) believed that genAI will contribute to improving patient care (Figure 3). However, opinions were more divided regarding genAI’s role in supporting empathetic care, with 34.3% in disagreement, 30.5% in agreement, and 35.3% remained neutral (Table 2). In the section for additional comments, 16.9% of respondents provided further input, with 26.5% expressing optimism about genAI’s potential benefits (Table 3). A common example cited was the reduction in time required for clinical documentation, highlighting genAI’s role in alleviating administrative burdens. For a more detailed breakdown of the responses, please refer to Supplementary Table 2.

**Table 2:** Physicians' perspectives on GenAI in clinical practice.

Statement	Strongly disagree No. (%)	Disagree No. (%)	Neutral No. (%)	Agree No. (%)	Strongly agree No. (%)
Physicians’ perceptions of GenAI					
GenAI will be a useful tool to increase the time physicians have for patient interaction. (*N*=289)	13 (4.50)	20 (6.92)	66 (22.84)	129 (44.64)	61 (21.11)
GenAI will be a useful tool to support empathetic care. (*N*=289)	29 (10.03)	70 (24.22)	102 (35.29)	58 (20.07)	30 (10.38)
GenAI and the patient-physician relationship					
Physicians' use of genAI will positively affect patients' trust in their clinical decisions. (*N*=281)	18 (6.41)	66 (23.49)	129 (45.91)	48 (17.08)	20 (7.12)
Physicians' use of genAI will enhance the development of a strong patient-physician relationship. (*N*=281)	18 (6.41)	68 (24.20)	126 (44.84)	50 (17.79)	19 (6.76)
Physicians' use of genAI can augment the placebo effect, by increasing patient confidence in their treatment. (*N*=281)	19 (6.76)	70 (24.91)	128 (45.55)	54 (16.93)	10 (3.56)
GenAI in clinical decision-making and patient interaction					
GenAI in clinical settings (eg., generative AI chatbots) can aid patients in selecting treatments. (*N*=263)	10 (3.80)	45 (17.11)	90 (34.22)	104 (39.54)	14 (5.32)
The placebo effect could be elicited through genAI-human interaction alone (eg., chatbots) ie., without the physician. (*N*=261)	17 (6.51)	68 (26.05)	105 (40.23)	63 (24.14)	8 (3.07)
Concerns regarding GenAI integration in patient care					
Are you concerned about the advances and integration of genAI into the healthcare system? (*N*=265)	21 (7.92)	63 (23.77)	100 (37.74)	61 (23.02)	20 (7.55)

**Table 3:** Physicians comment themes on GenAI in clinical care.

Section	No. (%)
Physicians’ perceptions of GenAI (*N*=49)	
Potential benefits and optimism	13 (26.53)
Potential usefulness, but not yet reliable	12 (24.49)
Uncertainty and lack of experience	11 (22.45)
Overall negative concerns and distrust in data quality	8 (16.33)
Specific concerns with empathy	3 (6.12)
Neutral opinion	2 (4.08)
GenAI and the patient-physician relationship (*N*=16)	
Overall negative concerns and distrust in data quality	5 (31.25)
Decreases patient trust, leads to general uncertainty over data quality	4 (25.00)
Uncertainty and lack of experience	3 (18.75)
Potential benefits and optimism	2 (12.50)
Mixed response	2 (12.50)
GenAI in clinical decision-making and patient interaction (*N*=11)	
Emphasizing importance of fostering patient relationship with a human physician	4 (36.36)
Uncertainty	3 (27.27)
General distrust of genAI	1 (9.09)
Disagrees with the proposed viewpoints/emphasis on placebo effect	1 (9.09)
GenAI not useful during patient visits, but still potential for use in healthcare	1 (9.09)
Emphasizing patient frustration, rather than anxiety	1 (9.09)
Concerns regarding genAI integration in patient care (*N*=110)	
Distrust and data concerns in genAI	47 (42.73)
Uncertainty	21 (19.09)
Potential clinical benefits and optimism	14 (12.73)
GenAI is a useful clinical tool, but does not replace human interaction	9 (8.18)
Potential usefulness, but not yet reliable in healthcare	9 (8.18)
Concern over genAI being implemented too quickly	5 (4.55)
Concern over decreasing human empathy/interaction	4 (3.64)
GenAI implementation is inevitable	1 (0.91)

### GenAI and the patient-physician relationship

This section examined physicians’ perceptions of genAI’s role in supporting patient trust, strengthening the physician-patient relationship, increasing patient confidence in treatments, and potentially augmenting the placebo effect. Physicians were divided on whether genAI would positively influence patient trust in clinical decisions, with 29.9% disagreeing, 24.2% agreeing, and 45.9% remaining neutral, indicating considerable uncertainty regarding genAI’s impact in this area. Similarly, opinions were mixed on genAI’s potential to strengthen the physician-patient relationship, as 44.8% remained neutral, 30.6% disagreed, and 24.6% agreed, suggesting a lack of clear consensus on its role in fostering clinician-patient interactions. A similar pattern emerged when assessing AI’s ability to enhance the placebo effect by increasing patient confidence in treatments. While 31.7% disagreed and 24.6% agreed, the majority (45.6%) remained neutral, reflecting uncertainty regarding whether genAI can effectively contribute to placebo effects (Table 2). Only 5.7% of respondents provided additional comments for this section, with the majority (56.3%) expressing concerns and uncertainty regarding data quality (Table 3).

### GenAI in clinical decision-making and patient interaction

This section explored genAI’s expanding role in clinical settings, particularly its potential to assist in treatment selection, replace aspects of physician-patient interaction, and engage directly with patients. Additionally, it examined physicians’ perspectives on genAI’s capacity to convey empathy, elicit placebo effects, and trigger the nocebo effect. Regarding AI’s role in treatment selection, 44.9% of physicians agreed that genAI technologies could aid patients in choosing treatments, while 20.9% disagreed, indicating a moderate level of support for genAI-assisted decision-making. There was a strong consensus on the role of genAI as a support tool rather than an autonomous decision-maker, with more than two-thirds (71.8%) of respondents agreeing that genAI should function primarily as an aid to physicians rather than engaging directly with patients. Opinions were divided on whether the placebo effect could be elicited solely through genAI-human interaction, without physician involvement. 40.2% remained neutral, 32.6% disagreed, and 27.2% agreed, highlighting uncertainty about genAI’s potential to induce placebo responses. There were some skepticism regarding perceptions on genAI’s capacity to convey empathy with 41.4% disagreeing and 25.9% agreeing. Moreover, the great majority of physicians (50.8%) agreed that genAI-human interactions could augment the nocebo effect by increasing patient anxiety about their treatments, highlighting potential concerns about genAI’s influence on treatment perception and patient well-being (Table 2; Figure 3). Only a small percentage of participants (4.2%) provided additional comments, with the majority emphasizing the importance of fostering the physician-patient relationship with a human doctor. Response categories are presented in Table 3 and original comments can be found in Supplementary Table 3.

### Concerns regarding GenAI integration in patient care

This last section explored physicians’ concerns about the use of genAI technologies in patient care. Opinions were divided, with 31.7% expressing no concern, 30.6% indicating worry, and 37.7% remaining neutral, suggesting that these respondents are either uncertain or have yet to form a definitive stance on the matter (Table 2). When asked participants to elaborate on their views, 41.5% provided written explanations that were categorized on Table 3. Among these responses, 42.7% reflected skepticism, distrust, or concerns about data reliability in genAI implementation. Physicians mentioned apprehensions regarding genAI accuracy, reliability, and its potential impact on clinical decision-making. Conversely, 12.7% highlighted potential benefits and expressed optimism about genAI’s role in patient care. A detailed breakdown of these categorized responses can be found in Supplementary Table 4.

## Discussion

This study examined how physicians at a major academic pediatric hospital in Boston are using and perceiving genAI in clinical care, with a focus on its implications for empathy, the physician-patient relatioship, and expectation-driven effects such as placebo and nocebo. Although LLM-based genAI technologies such as ChatGPT have only recently become available, our survey suggests that a substantial proportion of physicians have already begun integrating these tools into their clinical workflow. Within less than 2 years of its release, nearly two-thirds (65.2%) of physicians at BCH reported using LLM-based AI tools in practice, indicating a rapid adoption of technology. Consistent with previous work, our survey found that genAI adoption may be higher among younger physicians and men, mirroring broader trends in technology uptake across professions (Bychkov et al., 2024). However, not all surveys have identified this trend. This suggests that digital literacy and generational differences may shape early patterns of engagement with genAI. These findings point to the need for trained initiatives and inclusive implementation strategies that ensure all providers can benefit from technological advances, regardless of age or gender. Notably, the majority of the users (59.1%) reported relying on ChatGPT-4, the paid subscription version. This suggests a perceived value proposition that justifies a financial investment. The preference for a paid tool implies that clinicians recognize the added utility of advanced capabilities offered by newer LLM-based genAI tools. It also reflects a growing comfort among physicians with leveraging digital tools to optimize their practice (Ehrenfeld, 2023). This early and enthusiastic uptake underscores the urgency of understanding not only how these tools are used, but also how they may be shaping clinical reasoning, patient communication, and professional identity.

While this study was designed as an exploratory investigation, certain expectations could be inferred from prior literature. Based on earlier work, one might anticipate that physicians would express optimism about genAI’s potential to improve efficiency and aspects of patient care, yet remain skeptical about its capacity to convey empathy or deepen relational connection (Perry, 2023; Akingbola et al., 2024; Shuaib, 2024). Simirlarly, given the sensitivity of communication to framing and expectations, it might be anticipated that phsysicians would recognize genAI’s potential to elicit both placeboo and nocebo like responses (Smith, 2020; Faria et al., 2023). Our findings align with these general trends, showing enthusiasm for genAI practical benefits but uncertainty or concern regarding its emotional and psychosocial implications, while also highlighting areas where empirical evidence is still lacking. We note, however, that these reflections are *post hoc* and not pre-defined hypotheses, as the study was intentionally exploratory, taking the first steps to generate rather than test theoretical predictions. When asked about the primary purposes of genAI usage in clinical practice, physicians most commonly reported using genAI tools for writing letters or emails (55.8%), followed by clinical documentation (31.3%), and continuing medical education (21.6%). These findings are consistent with previous studies (Blease et al., 2024b; General Practitioners’ Experiences with Generative Artificial Intelligence in the UK: An Online Survey, 2025). For example, surveys in the UK investigating genAI adoption found that documentation was the leading usage (Blease et al., 2024b); one study conducted in 2025 found that of 1 in 4 general practitioners who used these tools, 35% reported using them for documentation purposes (General Practitioners’ Experiences with Generative Artificial Intelligence in the UK: An Online Survey, 2025). This pattern aligns with prior studies indicating that early genAI adoption in medicine is strongest in documentation-related tasks, and is driven by the desire to reduce administrative burden and free up time for direct patient care (Blease et al., 2020, 2022; Doraiswamy et al., 2020; Sharma B. et al., 2023; Shuaib, 2024). This also underscores the gap between genAI’s technical potential and its current application, highlighting an ongoing hesitancy to integrate genAI into more complex, nuanced aspects of care (Li and Abangbila, 2024), despite its growing use in patient communication, which involves relational and emotionally sensitive elements. The relatively modest uptake for clinical documentation (31.3%) may also point to lack of integration into existing electronic health records, or lingering concerns about data quality, privacy, and trustworthiness. Furthermore, the use of genAI for continuing medical education (21.6%) supports growing interest in these tools for professional development, enabling physicians to quickly access and synthesize complex information (Gavarkovs et al., 2025). Collectively, these results suggest that while genAI adoption is growing, its clinical role remains in the early stages, focused more on logistical support than on core therapeutic interactions. Regarding usage frequency, about one-fourth of physicians (25.9%) reported using genAI tools a few times per week and 16.8% reported daily or near-daily use, indicating a growing base of regular users, while nearly half (48.1%) reported infrequent use, defined as using genAI less than or only a few times per month. This disparity highlights that although genAI is beginning to take hold in clinical routines, its adoption remains uneven, likely shaped by differences in perceived utility, confidence, and lingering concerns (Chen and Liu, 2024).

Importantly, physicians appear to view genAI not only as a tool to improve efficiency, but also as a means to enhance the quality of patient care. A significant majority (65.7%) believed genAI could increase time available for direct patient interaction, a view supported by literature emphasizing how genAI may streamline documentation and reduce administrative load (Sharma B. et al., 2023; Mathur, 2024). Similarly, the majority of respondents (60.7%) believed that genAI will contribute to improving patient care, reflecting a general optimism among physicians about the technology’s potential to enhance clinical outcomes despite the reported uncertainties about its relational and ethical implications (Perry, 2023; Akingbola et al., 2024). However, perspectives were more divided when evaluating genAI’s ability to support empathy during patient-physician relationship. Approximately one-third of respondents disagreed with the notion that genAI could support empathetic care, while another third remained neutral. This ambivalence underscores ongoing skepticism about whether current genAI tools can replicate or augment emotional intelligence in clinical settings, a concern echoed by prior work (Perry, 2023). These findings reflect broader tensions in the literature between genAI’s growing functional capabilities and its perceived inability to engage with the moral and emotional complexity inherent to human caregiving (Kerasidou, 2021; Perry, 2023; Akingbola et al., 2024). In particular, research in psychiatric contexts has highlighted critical limitations in genAI’s capacity to recognize and respond appropriately to emotional distress, raising concerns about the reliability of simulated empathy in emotionally sensitive environments (De Freitas et al., 2024). At the same time, emerging evidence challenges the notion that genAI cannot convey empathy. Relatedly, questions must be asked about the appropriateness and consistency of clinicians’ responses to patients, which may sometimes fall short of full empathetic care (Gleichgerrcht and Decety, 2013). Recent studies suggest that user expectations play a pivotal role in shaping perceived empathy. When users are primed to view an genAI system as caring, they are more likely to interpret its responses as empathetic (Pataranutaporn et al., 2023). These contrasting findings underscore the complexity of interpreting genAI’s role in relational care and may help explain the uncertainty reflected in physicians’ responses in our survey.

In this survey physicians’ responses revealed significant uncertainty regarding genAI’s impact on the relational core of clinical care, including patient trust, the physician–patient relationship, and placebo-related mechanisms. Across all items in this section, nearly half of respondents selected neutral responses, indicating a lack of strong consensus or established experience with genAI in these more nuanced, psychosocial dimensions of care. This aligns with prior literature highlighting clinicians’ ambivalence toward genAI’s role in emotionally and ethically sensitive aspects of medicine (Singh et al., 2023; Blease et al., 2024a). Although clinicians acknowledge AI’s potential to support medical work, their views have been shown to be shaped by job-replacement anxiety, skepticism and limited knowledge (Weber et al., 2024). Trust, while central, yet unresolved in healthcare genAI adoption, has been shown to depend on perceptions of reliability, transparency, and human oversight, but can be undermined by concerns about accountability and unclear decision making (Markus et al., 2021; Afroogh et al., 2024; Nouis et al., 2025). The large portion of neutral responses in our survey suggest that many physicians have yet to form stable attitudes on whether genAI can enhance or erode trust in clinical care, underscoring the need for further empirical work in this area. Taken together, these results underscore how uncertainty continue to characterize medical professional views on genAI adoption, particularly when it comes to its more relational aspects. While genAI has been embraced for its operational efficiency, its potential to influence trust, therapeutic alliance, or expectation-based responses remains underexplored and, as these results suggest, underappreciated by many physicians. Notably, skepticism around genAI’s capacity to enhance placebo effects, an inherently relational and belief-driven phenomenon, further underscores the perception that these outcomes remain deeply tied to human interaction. These findings reinforce the need for clearer clinical frameworks and empirical evidence to guide physicians in understanding how genAI might shape not only clinical processes but also the subtler interpersonal dynamics that underpin patient outcomes.

Physicians’ responses regarding the role of genAI in clinical decision-making and patient interaction reflect a cautious yet pragmatic attitude. Nearly half (44.9%) of respondents agreed that genAI could assist patients in selecting treatments, suggesting moderate support for geAI-assisted decision-making. However, this support was clearly framed within a model of human oversight. A strong majority (71.8%) agreed that genAI should function primarily as a support tool for physicians, rather than as a direct communicator with patients. This finding aligns with prior literature emphasizing clinicians’ preference for maintaining human mediation in genAI-supported care (Festor et al., 2023). It also echoes concerns about preserving professional autonomy and the integrity of the physician–patient relationship in an increasingly automated environment (Funer and Wiesing, 2024). Physicians were more skeptical about genAI’s ability to perform relational functions. Over 40% of respondents disagreed that an genAI-patient interaction could convey empathy, while only 25.9% agreed, reinforcing prior critiques that current genAI systems lack the emotional nuance and contextual awareness necessary for truly empathetic engagement (Perry, 2023; Akingbola et al., 2024). The role of empathy in therapeutic relationships is well-established as a key driver of patient trust and outcomes (Hojat, 2016), and these results suggest that many clinicians remain unconvinced that these dimensions can be adequately simulated by generative models. Nonetheless, some studies suggest that among physicians with greater experience of genAI, the more likely they are to agree that these tools can be used to strengthen empathetic aspects of care (Blease et al., 2024a).

While 27.2% believed genAI-human interaction could elicit placebo effects, 32.6% disagreed and 40.2% remained neutral. This ambivalence likely reflects the complex and poorly understood nature of how patient expectations interact with technological interfaces. Prior studies have demonstrated that framing and belief can shape perceived empathy and therapeutic benefit from AI systems (Pataranutaporn et al., 2023), yet our findings suggest that physicians remain uncertain about how these dynamics operate without human involvement. By contrast, physicians expressed more definitive concern about genAI’s potential to trigger nocebo effects. Over half (50.8%) agreed that genAI-human interaction could increase patient anxiety, likely reflecting concerns about impersonal communication, lack of contextual sensitivity, and reduced capacity to offer reassurance. These concerns mirror recent literature cautioning that overreliance on automation, and dehumanized communication can erode trust and lead to depersonalized care (Akingbola et al., 2024). This supports previous results highlining the ethical risks of using genAI in emotionally charged settings without appropriate oversight. Physicians may have anticipated that patients will increasingly turn to genAI to interpret their medical records and decide when to seek care, potentially heightening anxiety. One study found that clinicians expected genAI to influence how patients engage with their health information (Blease et al., 2024c), including using these tools to understand their online records, and to decide when to seek medical attention. While it is unclear, physicians may have anticipated this, possibly fostering the view that patients might have negative expectations because of their inquires. Our findings suggest that the role of expectancy and its potential modulation through genAI-mediated communication is largely overlooked in conceptual models of healthcare communication. The observed physician ambivalence reflects not only empirical uncertainty but also a theoretical gap in how digital interfaces may alter the psychosocial mechanisms traditionally associated with human care.

Building on these concerns, the final section of the survey specifically explored physicians’ views on the advancement and integration of genAI technologies into the healthcare system. Responses were notably divided: 30.6% expressed concern, 31.7% reported no concern, and 37.7% remained neutral. This high degree of neutrality suggests that many physicians may still be uncertain about the broader implications of genAI, either due to limited exposure or the rapidly evolving nature of the technology. Among those who provided qualitative responses (41.5%), a substantial proportion (42.7%) voiced skepticism or distrust—particularly regarding data reliability, accuracy, and the potential impact of genAI on clinical decision-making. These concerns echo ongoing discussions in the literature about the risks of loss of clinical control, and the challenges of ensuring accountability in genAI-driven systems (Smith, 2020; Procter et al., 2022). Conversely, a smaller subset (12.7%) expressed optimism, highlighting potential benefits such as improved efficiency and decision support in complex clinical environments.

## Limitations

Several limitations should be considered when interpreting the findings of this study. First, the survey was developed for this project and was not subjected to formal psychometric validation, such as reliability or internal consistency testing. While face validity was established through expert review and piloting, the absence of standardized psychometric evaluation means that findings should be interpreted with caution. Future research should incorporate validated measures or conduct formal scale development to strengthen the robustness of conclusions. Second, the survey was conducted at a single academic pediatric hospital in Boston, which may limit the generalizability of the results to other clinical settings, specialties, or institutions with different levels of technological infrastructure or genAI exposure. Third, the response rate was modest (13%), and although consistent with other physician surveys (Faria et al., 2023), it introduces the potential for nonresponse bias. Physicians who chose to participate may have had stronger opinions or greater interest in genAI than those who did not respond. Fourth, the survey relied on self-reported data, which may be subject to recall bias or social desirability bias, particularly in questions assessing perceived impact on patient care or empathy. Fifth, given the rapidly evolving nature of genAI technologies and the absence of widespread clinical guidelines, respondents may have varied in their familiarity with specific tools or use cases, contributing to the high proportion of neutral responses observed in several domains. Finally, while the survey included open-ended comment fields, the format may not have captured the full depth of physicians’ reasoning or concerns. Future research incorporating qualitative interviews or focus groups could offer richer insights into how clinicians are thinking about genAI’s integration into both technical and relational aspects of care. Indeed, as genAI becomes increasingly integrated into health systems, assessing the ongoing perspectives of all stakeholders, not only physicians, will be essential in shaping ethical, effective, and patient-centered uses of these technologies. For example, patient perceptions of genAI’s role in healthcare communication also warrant close study. Qualitative and mixed-methods research could illuminate how patients interpret and emotionally respond to genAI-generated content, particularly in sensitive areas such as diagnosis, prognosis, and medication side effects. Additionally, research should examine whether trust, health literacy, and prior digital experience shape these responses. Comparative studies between human and genAI communication, in terms of tone, perceived empathy, and clarity, would help identify features that mitigate or exacerbate nocebo responses. Longitudinal designs may also be useful to assess how repeated exposure to genAI over time shapes expectations and health behaviors. Finally, future research could empirically investigate the extent to which genAI influences placebo and nocebo effects in clinical settings. This includes examining how genAI-generated health information affects patient expectations, symptom perception, and treatment outcomes. Studies could explore whether explanations or summaries produced by genAI modulate reassurance or anxiety differently than those given by human clinicians, and whether this varies across conditions or patient populations.

## Conclusion

Overall, the findings indicate that while physicians see promise in genAI’s ability to aid clinical reasoning and treatment decisions, they remain wary of its limitations in addressing the emotional, psychological, and relational aspects of care. The strong preference for using generative genAI as an assistive rather than autonomous tool reflects a desire to safeguard the human connection in medicine. This aligns with a growing body of research suggesting that physicians do not see genAI as a substitute for empathy, a view reinforced in our qualitative data where respondents highlighted the centrality of the physician–patient relationship. Importantly, our findings also show that physicians are uncertain about genAI’s ability to elicit placebo effects, many believe it could contribute to nocebo effects by increasing patient anxiety, highlighting concerns about how genAI mediated communication might inadvertently shape negative expectations. As genAI continues to evolve, understanding and addressing these relational concerns will be critical to ensuring its ethical and effective integration into clinical practice. This study contributes to a growing but still nascent field by highlighting that physicians, key decision-makers in how genAI is introduced into care, hold nuanced and sometimes ambivalent views about its psychosocial and relational impact. As genAI continues to advance, understanding how physicians navigate this evolving triadic relationship between physician, patient, and technology will be essential to ensuring that its integration enhances, not undermines, the human foundations of healthcare. Ensuring that medical education and ongoing training address both the capabilities and limitations of genAI will be essential to integrating these tools in ways that support, rather than compromise, human care.

## Data Availability

The original contributions presented in the study are included in the article/Supplementary material, further inquiries can be directed to the corresponding author.
