# Supplementary material for: Triadic relations in healthcare: surveying physicians’ perspectives on generative AI integration and its role on empathy, the placebo effect and patient care
Source: Front Psychol. 2025 Nov 12;16:1612215. doi: 10.3389/fpsyg.2025.1612215 (PMC12646921; doi:10.3389/fpsyg.2025.1612215)
Supplement: Supplementary file 1 [file Data_Sheet_1.docx]

# Info Sheet

Please read the information sheet below.

Informed Consent Form

Please review this information before participating.

Purpose of the research: Generative artificial intelligence (AI) is a cutting-edge technology that creates artificial agents capable of engaging through text (e.g., chatbots) and visuals (e.g., video/image). This survey aims to understand how doctors use and perceive AI's role in improving or possibly challenging their practice. It will explore physicians' views on how generative AI affects soft-skills in clinical practice, particularly in areas like empathy, patient-physician relationships, and the placebo effect. A current example of generative AI in healthcare is its use in analyzing medical images to improve diagnostic accuracy.

Time required: Participation in the entire study will take approximately five minutes to complete. Risks: There are no anticipated risks associated with participating in this study.

Benefits: The findings of this study will be shared with the academic community through a series of presentations and written reports, which will be made available to the subjects on request. Please contact Vanda Faria [(vanda.faria@gmail.com)](mailto:(vanda.faria@gmail.com) and we will send you summaries of the overall study results.

Confidentiality: Your participation in this study will remain confidential, and your identity will not be stored with your data. Email addresses were obtained through the Boston Children's Hospital listserv. We will collect and store your email, but it will not be linked to your answers in any way that could reveal your identity. Each data set will be represented by a unique number (e.g., "Participant #123"), so neither usernames nor email addresses will be visible to researchers. All identifying information will be removed before storage, and all data will be encrypted.

Participation and withdrawal: Your participation in this study is completely voluntary, and you may withdraw at any time by simply closing your web browser (no extra questions will be asked).

The nature and purpose of this research have been sufficiently explained and I agree to participate in this study. I understand that I am free to withdraw at any time without incurring any penalty. I have read and understood the consent information and desire to participate in this study.

Please save or print a copy of this page for your records. [Attachment: "Informed Consent Form.docx"]

# Experience and Usage of Generative AI in Clinical Settings

Please complete the survey below. Thank you!

What kind of generative artificial intelligence (AI) tools do you use?


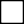
 ChatGPT3.5
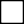
 ChatGPT4
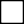
 Copilot


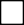
Gemini
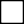
 BING AI
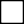
 BIOGPT


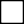
 Jasper


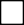
 Abridge's Galen
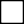
 Cloud


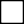
 Perplexity
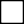
 Nuance DAX
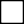
 None


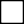
 Others

If "Others," please specify:

For what purpose do you use them?


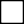
 Diagnostic assistance
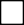
 Patient management
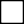
 Treatment planning


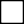
 Clinical documentation
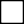
 Patient education


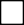
 Summarizing patient data from electronic health records
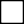
 Writing letters/emails


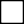
 Enhancing communication with patients
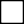
 Mental health support


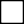
 Continued education
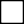
 Managing calendars
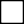
 Others

If "Others," please specify:

How often do you use generative AI tools in your regular work?

Never


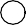

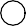

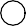

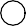

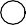

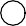

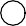


Less than once a month A few times a month

A few times a week Almost every day Every-day

Other

If "Other," please specify:

# Anticipated Impact of Generative AI on Physicians' Empathy and Patient Care

**The following items request your level of agreement concerning the support provided by**

**Generative AI in delivering empathy and patient care:**

1. Generative AI will be a useful tool to increase the time physicians have for patient interaction.

1 = strongly

disagree


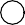


2 = disagree 3 = neutral 4 = agree 5 = strongly

agree


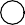

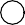

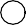

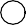


1.
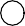

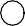

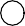

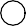

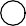
Generative AI technologies will be a useful tool to support empathetic care.
2.
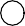

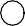

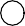

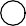

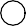
In general, generative AI technologies will improve patient care.
3. Additional comments:

# Anticipated Impact of Generative AI as a supportive tool for the Physician on the Patient-Physician Relationship

**The following items request your level of agreement concerning the role of generative AI in**

**supporting Patient-Physician relationships. The items refer to doctors' use of these tools.**

1. Physicians' use of generative AI technologies will positively affect patients' trust in their clinical decisions.

1 = strongly

disagree


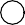


2 = disagree 3 = neutral 4 = agree 5 = strongly

agree


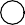

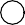

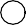

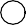


1.
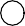

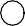

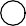

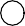

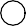
Physicians' use of generative AI technologies will enhance the development of a strong patient-physician relationship.
2.
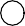

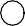

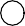

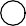

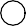
The placebo effect refers to a beneficial health outcome caused by a patient's positive treatment expectations. Physicians' use of Generative AI technologies can augment the placebo effect, by increasing patient confidence in their treatment.
3. Additional comments:

# Anticipated impact of Generative AI-human interaction in clinical settings

**The following items request your level of agreement concerning Generative AI adopting a more forefront role in clinical settings. The items refer to generative AI as potentially**

**replacing physician interactions.**

1. AI technologies in clinical settings (eg., generative AI chatbots) can aid patients in selecting treatments.

1 = strongly

disagree


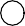


2 = disagree 3 = neutral 4 = agree 5 = strongly

agree


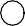

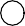

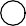

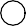


1.
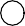

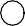

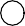

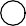

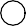
AI technologies in clinical settings (eg., generative AI chatbots) should only serve as support aids to physicians rather than directly interacting with patients.
2.
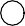

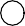

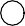

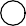

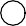
The placebo effect, defined as a beneficial health outcome caused by a patient's treatment expectation, could be elicited through AI-human interaction alone (eg., chatbots) ie., without the physician.
3.
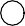

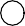

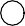

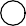

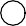
AI-human interactions in clinical settings, through generative AI chatbots, can convey empathy.
4.
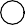

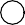

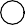

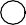

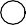
The nocebo effect reflects a negative health outcome caused by a patient's negative treatment expectations.

AI-human interactions in clinical settings, through Generative AI Chatbots, can augment the nocebo effect by causing patients anxiety about their treatment.

1. Additional comments:

# General

1. Are you concerned about the advances and integration of generative AI technologies into the healthcare system?

1 = Not at all worried 2


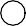

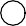

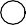

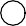

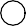


3

4

5 = Extremely worried

1. Please explain your answer:

# Demographics

Age:

Gender:

Man Woman


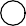

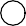

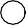

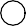


Prefer not to say Not listed above

If "Not listed above," please specify:

Medical Specialty:

Allergy and Immunology Anesthesiology Cardiology


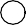

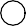

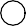

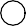


Dermatology Emergency Medicine Endocrinology Gastroenterology Hematology Hospital Medicine Infectious Diseases Intensive Care Neonatology Nephrology Neurology

Oncology Ophthalmology Orthopedics Otolaryngology Pediatrics Primary Care Psychiatry Pulmonology Rheumatology Surgery Urology

Other

If "Other," please specify:

Years in practice:
